# Feasibility of a new soft ankle exoskeleton on people with dropfoot post-stroke

**DOI:** 10.1017/wtc.2026.10043

**Published:** 2026-06-08

**Authors:** Xiaochen Zhang, Axel Fredriksen, Elena M. Gutierrez-Farewik, Susanne Palmcrantz

**Affiliations:** 1 KTH MoveAbility, the Department of Engineering Mechanics, KTH Royal Institute of Technology, Sweden; 2 Department of Clinical Sciences, Danderyd Hospital, Karolinska Institutet, Sweden; 3 Department of Women’s and Children’s Health, Karolinska Institutet, Sweden

**Keywords:** assistive device, biomechanics, gait impairment, soft robotics

## Abstract

Dropfoot gait pattern during walking commonly persists after stroke and is often associated with muscle weakness and pathological muscle activation. Exoskeletons have demonstrated the potential to improve mobility in people with neurological conditions. We have developed a novel soft ankle exoskeleton and shown its ability to correct simulated dropfoot and excessive inversion in nondisabled people. In this study, we evaluate its feasibility in five persons with chronic stroke and dropfoot gait patterns. 3D gait analysis was performed in three conditions: walking with only shoes, with the exoskeleton unpowered, and powered. Foot and ankle kinematics and step length asymmetry were evaluated. The participants also reported satisfaction with QUEST 2.0 and a study-specific questionnaire. Compared with only shoes, the powered exoskeleton partially corrected dropfoot by increasing dorsiflexion angle and foot clearance height in swing, facilitating heel contact, neutralizing ankle inversion, and increasing step length symmetry slightly. The participants expressed satisfaction with the exoskeleton’s effectiveness, though some comfort-related issues were identified. This feasibility study suggests that the exoskeleton prototype can improve dropfoot gait patterns and be accepted by individuals in the chronic stage after a stroke.

## 1. Introduction

Impaired ability to lift and control the foot during walking, often referred to as dropfoot, is a common consequence of stroke (Kluding et al., 2013). This condition is typically associated with muscle weakness and abnormal motor control. When spasticity of the tibialis posterior muscle is present, dropfoot can be further complicated by excessive ankle inversion (Beyaert et al., 2015). To compensate for dropfoot during swing, individuals may adopt strategies to increase foot clearance height, such as pelvic hiking and hip circumduction (Kerrigan et al., 2000; Farris et al., 2015). There is an increased risk for tripping or falling in dropfoot gait, as the more affected foot may drag on the ground and be prone to stumbling on obstacles (Perry and Burnfield, 2010). Stability in ground contact phases is also compromised by excessive ankle inversion (Konradsen and Voigt, 2002). Addressing dropfoot is thus essential for improving activities of daily life and promoting independence and confidence (Awad et al., 2017; Yeung et al., 2021).

Orthotic devices such as ankle-foot orthoses (AFOs) are useful and effective solutions for rehabilitation and for daily assistance after a stroke or other neurological injury. By providing support to lift the foot in swing and facilitate initial heel contact, AFOs have demonstrated success in compensating for dropfoot gait pattern but shown limited function in restoring gait adaptability across varying environments (Van Swigchem et al., 2014).

Active wearable devices, such as exoskeletons, have demonstrated potential to improve mobility in people with neurological conditions by providing active assistance (Louie and Eng, 2016). Among them, rigid exoskeletons, including powered AFOs, have shown promising improvements in dropfoot gait patterns (Blaya and Herr, 2004), but several limitations exist, including misalignment between the device’s hinge joint and the user’s ankle joint (Meier et al., 2023) and low muscle activation resulting from the high level of assistance (Sloot et al., 2023). In recent years, soft exoskeletons and exosuits have emerged as promising alternatives. By implementing softer or more compliant materials and new force transmission methods, including pneumatic systems (Park et al., 2014; Thalman et al., 2019; Kim et al., 2020; Xia et al., 2020) and cable-driven transmissions (Bae et al., 2018; Lerner et al., 2018; de Miguel-Fernández et al., 2022), soft exoskeletons offer several advantages and user-friendly features over rigid devices, including lower weight (Noronha et al., 2022) and better weight distribution, and can encourage some muscle activation in assisted muscles (Washabaugh et al., 2018; Sloot et al., 2023), indicating more user engagement in movement. While a few recently developed exoskeletons have targeted gait in people with dropfoot, few have also considered frontal plane malalignments (Zhong et al., 2021). To the best of our knowledge, none have been tested on persons with gait deviations in both sagittal and frontal planes.

We have developed a soft ankle exoskeleton prototype that can assist in dorsiflexion and eversion simultaneously, aimed to increase foot clearance in swing and to position the foot segment for initial heel contact, that is, with elevation in the sagittal plane and a neutral angle in the frontal plane (Zhang et al., 2024). We described the device and the biplanar control in a previous study (Zhang et al., 2024), and demonstrated its efficacy to improve foot clearance height in swing, sagittal, and frontal plane foot position in initial contact, and step length symmetry in a small group of nondisabled people with simulated dropfoot gait pattern.

Motivated by these findings, the purpose of this study is to evaluate the exoskeleton’s feasibility in a sample of participants in a chronic phase after stroke who walk with a dropfoot gait pattern, with or without excessive ankle inversion. We explore if and how the exoskeleton impacts gait patterns, specifically during swing and at initial foot contact, and assess user-reported satisfaction related to the exoskeleton’s functionality and usability.

## 2. Materials and methods

### 2.1. Ankle exoskeleton

#### 2.1.1. Hardware

**Figure 1.**
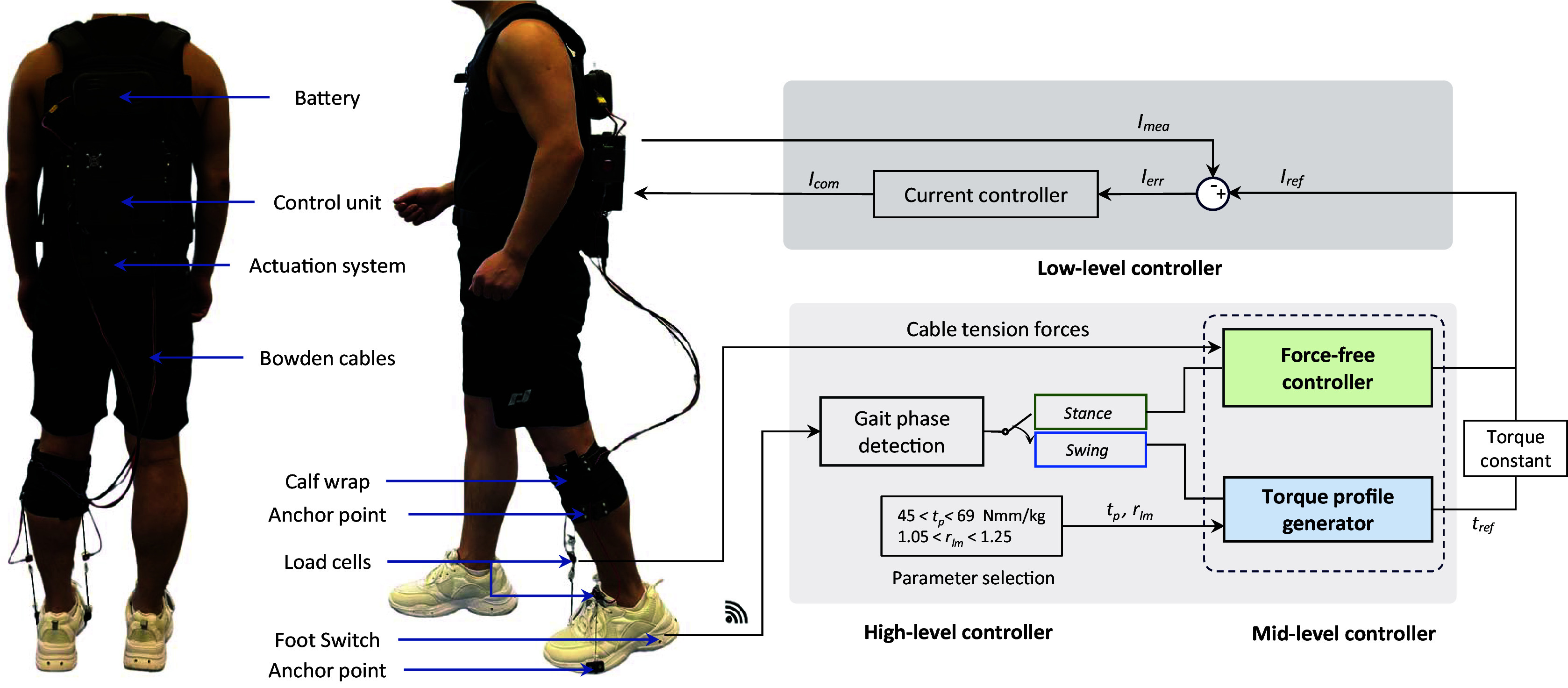
Cable-driven ankle exoskeleton for biplanar assistance. (a) The exoskeleton hardware consists of a backpack that houses the actuation model, control unit, and battery, two Bowden cables that span from the actuator to the shoe forefoot, a textile calf wrap, and sensors. (b) The three-level controller framework. The high-level controller detects gait phases. The two-mode mid-level controller consists of a current profile generator and a force-free controller, with mode switching based on gait phase detection results. The low-level controller drives the actuators to track the desired profiles generated by the mid-level controller. Figure 1. long description.

The ankle exoskeleton used in this study was described in a recent study (Figure 1) (Zhang et al., 2024). The total weight of the exoskeleton is 3.6 kg, including a backpack section, two Bowden cables, textile components, and sensors.

The backpack houses the actuation module, control unit, and battery. The actuation module features two brushless DC motors (90 W, Maxon, Switzerland), planetary gearboxes (123:1, Maxon, Switzerland), 3D-printed pulleys (14-mm radius), and Bowden cables. With a maximum motor torque of 45.1 mNm, each cable can generate up to a maximum of 396 *N* retraction force. A microcomputer (Raspberry Pi 4B, Raspberry Pi Foundation, UK) was used to control the actuators by sending control signals to motor drivers (EPOS4, Maxon Inc., Switzerland). A 24 V battery powers the actuation module directly and the microcomputer via a 24 V–5 V DC–DC converter.

Through the pulley, one end of the Bowden cable is attached to the actuator, and the other end spans through an outer cable housing anchored to the calf wrap and attaches to an anchor point on the shoe forefoot. One motor controls the cable attached to the medial side of the shoe forefoot, referred to as “medial cable” and “medial motor” here, and the other, to the lateral side, referred to as “lateral cable” and “lateral motor” here. When the actuation system is activated, the Bowden cable retracts and shortens the distance between the anchor points on the calf wrap and on the shoe, producing motion in the user’s ankle joint.

A foot switch (Cometa, Italy), with four transducers, is attached to the bottom of the shoe (on the paretic side) to detect gait phase. Two load cells (Futek, USA) in series with the Bowden cables measure cable tension and are used in the force-free controller that compensates for inherent inertia and resistance in the device and structure.

#### 2.1.2. Controller

The controller was designed to deliver assistance on the paretic side during the duration beginning at toe-off and ending just after foot contact.

A three-level hierarchical controller that consists of high-level, mid-level, and low-level controllers was designed in this study. The high- and mid-level controls were performed by the microcomputer, and the low-level control was embedded in the motor driver.

The high-level controller, functioning as a perception block, detects stance and swing phases from the signals transmitted from foot switches via the receiver and TCP/IP protocol.

**Figure 2.**
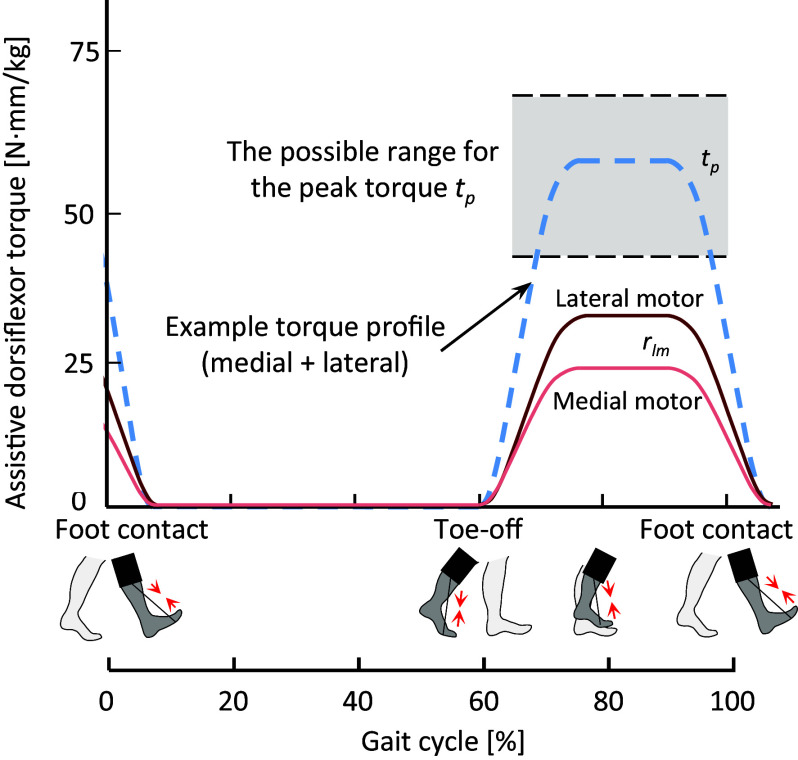
Example of the assistive dorsiflexion torque profiles. The pink line illustrates an example torque profile for the medial motor, and the red line for the lateral motor, determined from the torque ratio between the lateral and medial motors, $r_{lm}$ . The gray shaded area illustrates the possible range for the peak torque $t_{p}$ , that is, the sum torque of medial and lateral motors (the blue dashed line illustrates an example). Figure 2. long description.

A two-mode controller was implemented in the mid-level controller, serving as a profile generator. In the torque profile generator, the dorsiflexion assistance torque profiles, which provide lift to counteract dropfoot, were parameterized in the swing phase, as shown in Figure 2. The profiles were defined by two primary variables: the torque magnitude of the sum of the two motors ( $t_{p}$ ), and the torque ratio between the lateral and medial motors ( $r_{lm}$ ). Importantly, the torque ratio $r_{lm}$ modulates the differential assistance applied to the ankle, thereby also controlling the frontal plane motion, that is, inversion and eversion, of the foot. The swing phase duration was estimated as the average swing phase duration during the previous three steps. The ramp ascent and descent time of the torque profile occupied around 25% of the swing phase duration each. The setting of the force-free controller followed the same setting as described in Zhang et al. (2024), and generates the profiles to compensate in the stance phase for cable friction and actuator inertia during ankle plantarflexion, specifically the preswing phase.

The low-level control was implemented in the motor driver to control the motors and track the profiles set in the mid-level control.

### 2.2. Participant recruitment

Eligible participants fulfilled the following inclusion criteria: age between 20 and 60 years old, at least 1 year post stroke (chronic stage), impaired dorsiflexion function, able to walk independently with or without an AFO and handheld assistance, able to control excessive supination while walking on a flat surface with shoes but without an AFO, indicating no high injury risk from a fall or sprained ankle, and no known other injury or pathology in the lower limbs. Exclusion criteria were dependence on a walker or manual support while walking.

Five participants with chronic stroke (2 M/3F (median [min, max]) height: 168 [159, 184] cm, weight: 93 [73, 100] kg, age: 46 [42, 58] yrs) were recruited to and participated in this feasibility study (Table 1). The Swedish Ethical Review Authority approved this experiment (Dnr. 2023-02891-01). The participants provided informed written consent before the experiment.

**Table 1.** Participant information Table 1. long description.

ID	Paretic side	Time since stroke onset (months)	Walking aids	FMA-LE		MAS	RoM-Ank
				Motor function	Sensory function		
P01	Left	21	None	26	7	(0, 0, 0)	(0, 30)
P02	Left	60	AFO	21	7	(3, 3, 1)	(−5, 30)
P03	Left	58	AFO	25	9	(2, 0, 0)	(−5, 45)
P04	Left	24	AFO	25	9	(0, 0, 0)	(20, 30)
P05	Right	39	Dictus band	29	12	(0, 2, 0)	(5, 60)

*Note:* FMA-LE, Fugl–Meyer Assessment lower extremities, motor function (score 0–34, wherein the maximum score indicates no impairment) and sensory function (score 0–12, wherein the maximum score indicates no impairments); MAS, Modified Ashworth Scale (score 0–5, where 0 indicates no spasticity) describing spasticity of the soleus, gastrocnemius and ankle supinator muscles on the paretic side; no spasticity was observed in the other lower limb muscle groups (hip adductors, knee flexors, and knee extensors); RoM-Ank, Passive range of motion of ankle joint when knee joint angle is 0° (max dorsifleion, max plantarflexion).

Clinical assessments were performed by a licensed physiotherapist during the experiment. Assessments included the Fugl–Meyer assessment of motor recovery after stroke for the lower extremity (FMA-LE). In this study, the sub-score for motor function (score 0–34, wherein the maximum score indicates no impairment) and for sensory functions related to the perception of touch and proprioception (score 0–12, wherein the maximum score indicates no impairment) were included. Passive range of motion at the ankle joint was measured using a goniometer with a knee joint angle of 0°. The Modified Ashworth Scale (MAS) was used to assess spasticity of the lower extremity (score 0–5, wherein 0 indicates no spasticity), including the hip adductors, knee flexors, knee extensors, ankle plantarflexors (soleus and gastrocnemius), and ankle supinators. Spasticity scores for the ankle plantarflexors (soleus and gastrocnemius) and ankle supinators are presented in Table 1. No spasticity was observed in the other muscle groups.

### 2.3. Experimental protocol

Experiments were conducted at the Promobilia MoveAbility Lab, KTH Royal Institute of Technology, Stockholm. The lab was equipped with a 10-camera motion capture system (Vicon V16, UK) and a 10-m-long walkway. Forty-three reflective markers were placed on the trunk and lower limbs according to a common model (CGM 2.4), and their trajectories were recorded at a sampling frequency of 100 Hz. One surface electromyography (EMG) sensor (myon nano, Cometa, Italy) was placed over the tibialis anterior (TA) of the paretic side according to SENIAM recommendations (Hermens et al., 1999).

All participants walked on the laboratory walkway at their preferred speeds with standardized athletic shoes (PUMA, Unisex Flyer Runner), in four conditions:

- NoExo: with shoes. Each participant’s baseline gait was defined by the NoExo condition.
- Familiarization: with the exoskeleton and a few different assistive profiles. Participants walked with the powered exoskeleton for 30 min to adapt to the exoskeleton. No explicit instructions were given, so as to encourage participants’ natural response to the exoskeleton.
- UnpowExo: with the exoskeleton structure, but unpowered and slackened cable. In this condition, the influence of the exoskeleton structure independent of the assistance was evaluated, such as the weight and overall physical interface.
- PowExo: with the powered exoskeleton. Various swing phase assistance profiles were tested, parameterized by [ $t_{p}$ , $r_{lm}$ ] (sum torque magnitude of the two motors, the torque ratio between the lateral and medial motors), based on each participant’s swing phase duration. Assistance profiles in the range of [57 ± 12 Nmm/kg, 1.15 ± 0.1] were tested, with the summed torque as per the suggestion (Yeung et al., 2021). Ten assistance profiles were generated within this range, according to a uniform distribution. In the preswing phase, a force-free controller (Zhang et al., 2024) was implemented to compensate for the resistance in the device during the pre-swing phase. Participants walked along the 10-m walkway twice with each assistance profile, with ~1-min intervals for rest between different profiles.

### 2.4. Data collection and analysis

#### 2.4.1. Kinematics data

Kinematics during gait were analyzed via the CGM 2.4 inverse kinematics tool in Vicon Nexus. Step length was defined as the distance between the ipsilateral and the contralateral heel markers at initial contact in the direction of walking.

**Figure 3.**
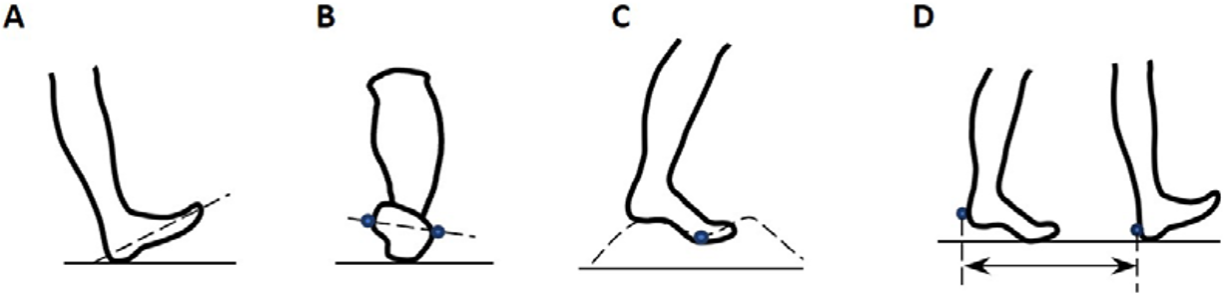
(a) Foot inclination angle at initial contact. (b) Foot inversion angle at initial contact. (c) Foot clearance during the swing phase. (d) Step length measured by heel markers.

Several key metrics of ankle and foot kinematics related to dropfoot gait patterns and exoskeleton efficacy were selected for analysis (Figure 3). These include:

- Ankle dorsiflexion angle at initial contact
- Foot inversion angle at initial contact
- Foot segment inclination angle, or foot-to-floor angle, at initial contact
- Average foot clearance height in swing, defined as the vertical difference of the fifth metatarsal head marker between stance and swing
- Peak ankle dorsiflexion angle in swing
- Peak foot inversion angle in swing
- Step length asymmetry index ${ASI}_{SL}$ , defined as the step length difference divided by the average, as a percent (Eq. 1) (Bae et al., 2015; Yul Shin et al., 2020).

(1) $${ASI}_{SL}=\left|\frac{L_{\mathrm{step},p}-L_{\mathrm{step},\mathrm{np}}}{0.5\cdot (L_{\mathrm{step},p}+L_{\mathrm{step},\mathrm{np})}}\right|\times 100\%$$

where $L_{\mathrm{step},p}$ and $L_{\mathrm{step},\mathrm{np}}$ are the step lengths of the paretic and nonparetic sides, respectively.

#### 2.4.2. EMG data

EMG signals were band-pass-filtered (20–400 Hz, Butterworth 4th order), rectified, and low-pass-filtered (4 Hz, Butterworth 4th order). All negative values after the low-pass filter were replaced by zero. For each participant, the EMG signal of TA was normalized to its maximum during the whole session.

The mean normalized TA activation during the swing phase was computed for all conditions.

#### 2.4.3. Subjective assessment

To evaluate the subjective user experience and the usability of the exoskeleton, participants reported their satisfaction based on two questionnaires.

The first questionnaire is the Quebec User Evaluation of Satisfaction with Assistive Technology 2.0 (QUEST 2.0) (Demers et al., 2000). In the current study, five items related to everyday use experience were included: dimensions, weight, stability and security, comfort, and effectiveness, and participants reported their satisfaction on a scale from 1: “not satisfied at all” to 5: “very satisfied.” The scores can thus be interpreted as dissatisfied (1–2.5), neutral (2.5–3.5), and satisfied (3.5–5).

A second study-specific questionnaire was developed to gain insight into the perceived relative advantages and experience of the exoskeleton. It included six questions related to (1) perceived ease while walking, (2) potential discomfort, (3) skin irritation, (4) pain, (5) perceived benefits of the exoskeleton, and (6) perceived benefits of their habitual orthoses. Responses were scored on a scale from 0: “not at all” to 10: “to the maximum extent.” The scores for items 1 and 5 can thus be interpreted as negative experience (0–4), neutral (4–7), and positive experience (7–10). The scores for items 2, 3, and 4 are inverse: positive experience (1–4), neutral (4–7), and negative experience (7–10).

#### 2.4.4. Statistical analyses and minimal detectable change comparisons

The differences in metrics between the NoExo, UnpowExo, and PowExo conditions were first evaluated using repeated-measures ANOVA. Post-hoc paired *t*-tests were then conducted to test whether differences in gait metrics exist between conditions (PowExo vs. NoExo, PowExo vs. UnpowExo, and UnpowExo vs. NoExo). The level of significance for all tests was set at α=0.05 .

To evaluate whether observed improvements were meaningful, changes in ankle joint angle during swing and at initial contact were compared to the reported minimal intra-session detectable change (MDC) in individuals post-stroke (Kesar et al., 2011).

## 3. Results

### 3.1. Biomechanical responses

**Figure 4.**
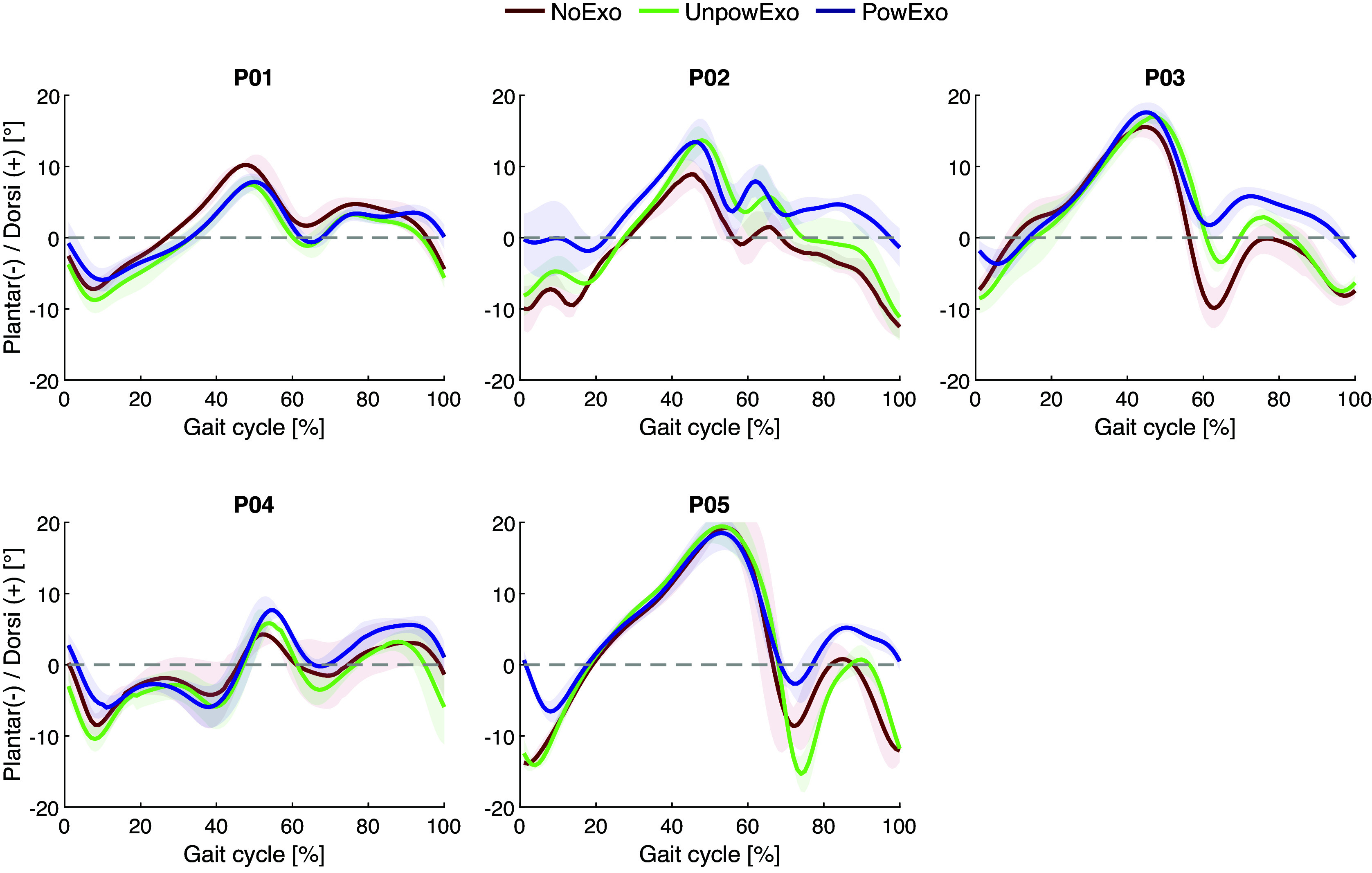
Ankle joint kinematics with the powered (PowExo) and unpowered (UnpowExo) exoskeleton and with only shoes (NoExo) in five participants. Figure 4. long description.

Compared with the NoExo condition, dropfoot was partially corrected in the PowExo condition (ankle joint kinematics observed in all five participants are shown in Figure 4). The ankle angle was closer to a neutral position in the swing phase, leading to an increase in ankle dorsiflexion angle at initial contact, and a heel rocker movement was subsequently observed, especially for P03 and P05. The plantarflexion angle in the pre-swing phase was slightly decreased, which is caused by the residual resistance in the system during push-off.

**Figure 5.**
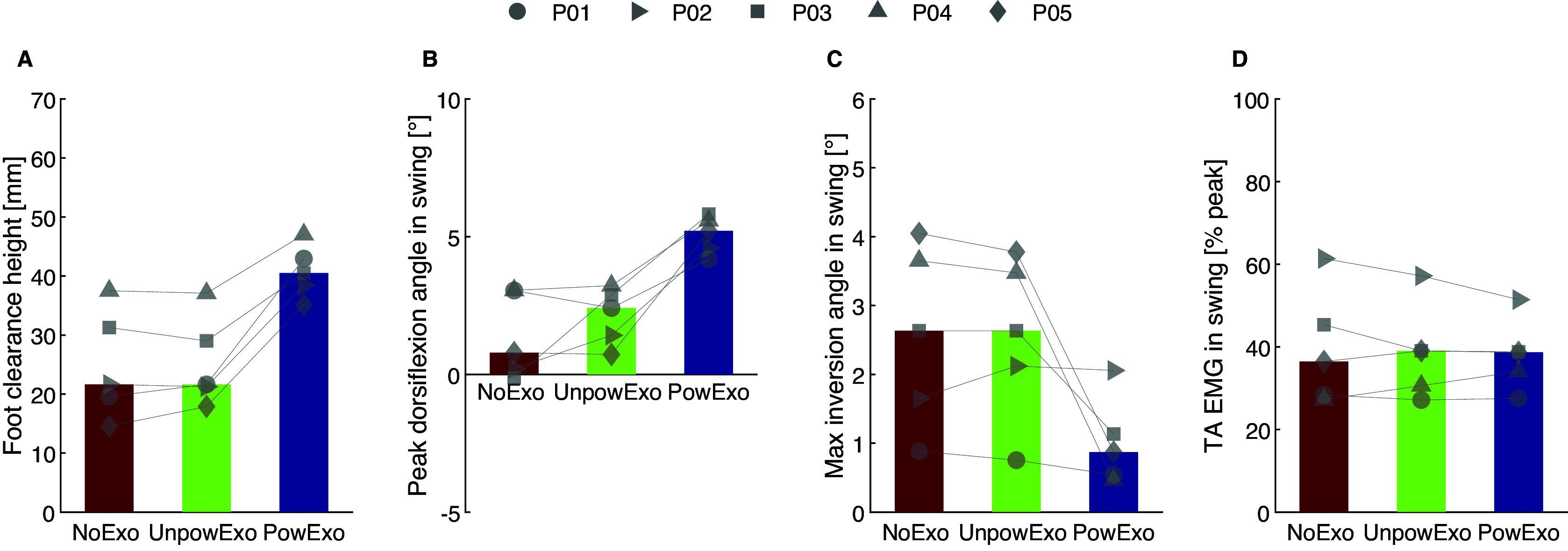
Swing phase gait metrics in the five participants and three conditions: NoExo, UnpowExo, and PowExo. The bar plots depict median values across the participants, and individual markers depict each participant’s measured data. (a) Foot clearance height. (b) Peak dorsiflexion angle. (c) Maximum inversion angle. (d) Average tibialis anterior normalized EMG. Figure 5. long description.

With the powered exoskeleton, in the swing phase, all five participants showed higher foot clearance height and higher peak dorsiflexion angle, compared to NoExo and UnpowExo conditions (Figure 5); foot clearance height increased by 16.8 (9.2, 20.6) mm (*p* = 0.007, values are presented as median [minimum, maximum] throughout the Results section) and peak dorsiflexion angle increased by 4.4 (1.1, 5.9)° compared to NoExo (*p* = 0.039), considerably higher than the reported 0.9° MDC (Kesar et al., 2011). Maximum inversion angle in swing decreased in the 3/5 participants with the most inversion angle in the NoExo condition, and was largely unchanged in the other 2/5 participants. Only slight changes were observed in the tibialis anterior muscle activity among the three conditions (*p* = 0.397).

Compared to NoExo, in the UnpowExo condition, ankle angles and foot clearance height were largely unchanged (*p* = 0.559).

**Figure 6.**
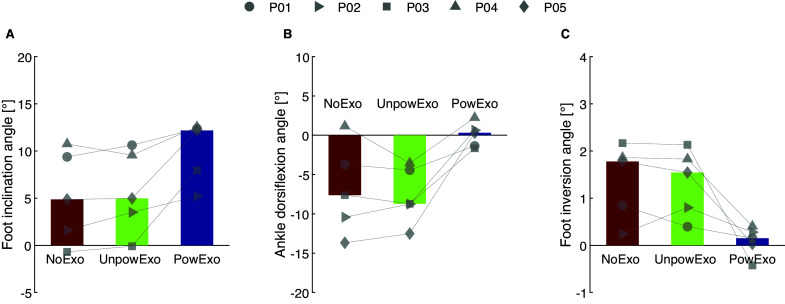
Foot and ankle positions at initial contact in the five participants and three conditions: NoExo, UnpowExo, and PowExo. The bar plots depict median values across the participants, and individual markers depict each participant’s measured data. (a) Foot inclination angle. (b) Ankle dorsiflexion angle. (c) Foot inversion angle. Figure 6. long description.

With the powered exoskeleton, at initial contact, the foot inclination angle was higher and more dorsiflexed in all five participants (Figure 6); average difference in foot inclination angle was 3.0 [0.2, 8.6]° (*p* = 0.009) and in ankle dorsiflexion, 5.9 [1.1, 13.9]° (*p* = 0.114), again considerably higher than the reported MDC of 0.9°. Ankle inversion angle at initial contact decreased in the same 3/5 participants with the most inversion angle in the NoExo condition, and was largely unchanged in the other 2/5 participants.

Compared to NoExo, in the UnpowExo condition, foot inclination angle and ankle sagittal and frontal plane angles were largely unchanged (all *p* > 0.05).

**Figure 7.**
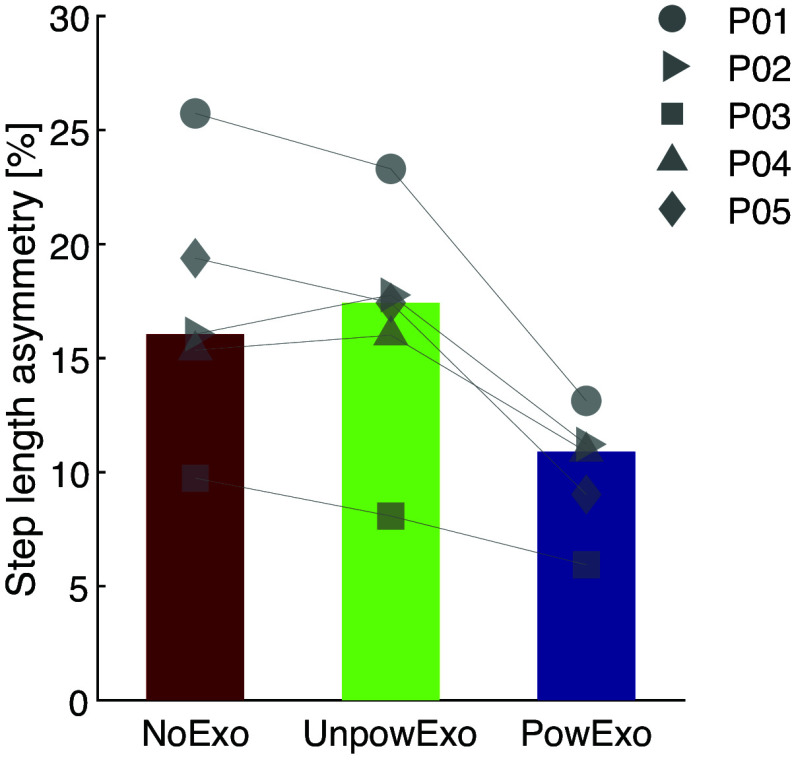
Step length asymmetry in the five participants and three conditions: NoExo, UnpowExo, and PowExo. The bar plots depict median values across the participants, and individual markers depict each participant’s measured data. Figure 7. long description.

Compared to NoExo, in PowExo, step length asymmetry decreased in all five participants by a median of 4.8 (3.8, 10.3)% (*p* = 0.013), as shown in Figure 7. Compared to NoExo, in the UnpowExo condition, step length asymmetry increased slightly, though not significantly (*p* > 0.05).

### 3.2. Questionnaire responses

**Figure 8.**
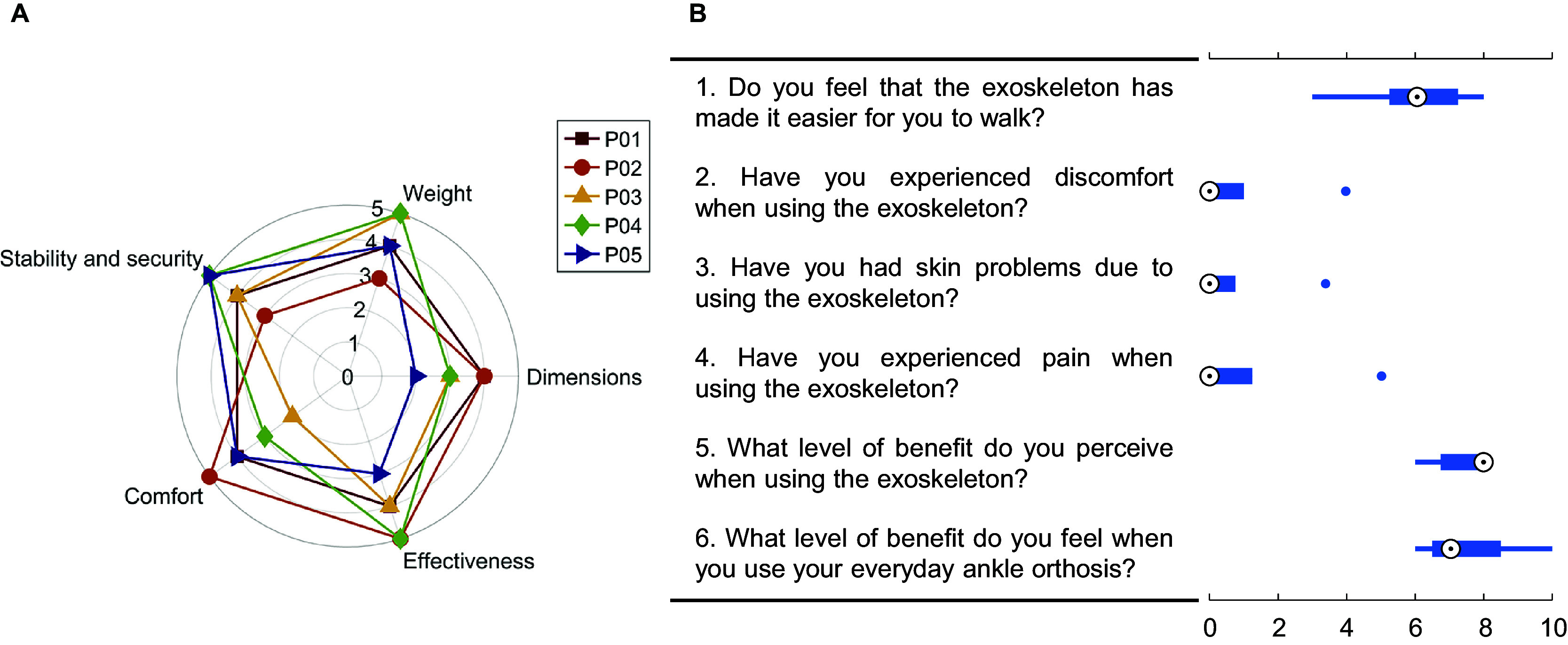
Scores from the two questionnaires on participants’ satisfaction with the exoskeleton. (a) Questionnaire 1 – QUEST 2.0: Participants rated across five dimensions: stability, weight, comfort, effectiveness, and dimensions. (b) Questionnaire 2: The study-specific questionnaire reported the discomfort-related issues and the perceived benefits. In the box plots, the white circle is the median, and the bounds of boxes represent the 25th and 75th percentile quartiles. Figure 8. long description.

In the QUEST 2.0 questionnaire, participants expressed an overall score of 3.88, indicating satisfaction (Figure 8). Specifically, participants reported satisfaction with the device’s weight (4 [3, 5]), stability and security (4 [3, 5]), effectiveness (4 [3, 5]), and comfort (4 [2, 5]), and neutral satisfaction with the dimensions (3 [2, 4]).

In the second questionnaire, participants expressed mostly positive experiences regarding comfort (item 2, median score 0 [0, 4]), skin irritation (item 3, median score 0 [0, 3]), and pain (item 4, median score 0 [0, 5]).

One participant who initially scored 2 (out of 5) for comfort on the QUEST 2.0 reported scores of 4, 3, and 5 for items 2, 3, and 4, respectively (blue dots in Figure 8B). None of the other four participants reported any discomfort issues. A neutral experience was reported on the question of whether participants perceived it was easier to walk with the exoskeleton (6 [3, 8]). Participants scored the perceived benefits from the exoskeleton and from their regular orthoses very similarly, with scores of 8 [6, 8] and 7 [6, 10], respectively.

## 4. Discussion

In this feasibility study, we investigated both objective gait responses and subjective perception of a newly designed ankle exoskeleton aimed at assisting ankle joint movements associated with dropfoot gait patterns, in a pilot group of five individuals post-stroke. The exoskeleton’s assistance shows promise to improve nearly all targeted gait metrics, including foot clearance and ankle position in swing, and foot inclination and ankle sagittal and frontal plane angles at foot clearance. Changes in dorsiflexion angle with the exoskeleton exceed the reported intra-session MDC in dorsiflexion of 0.9° (Kesar et al., 2011). Step length asymmetry was also improved in this pilot group, indicating a holistic improvement in gait kinematics. Participants reported overall satisfaction with the device.

Specifically, the assistance initiated at foot-off, and increased ankle dorsiflexion during mid- to late swing, similar to previously reported assistive ankle exoskeletons for dropfoot (Bae et al., 2018). However, to the best of our knowledge, this is the first reported soft exoskeleton to also simultaneously correct ankle motion in the frontal plane during swing. The increased foot clearance height can potentially reduce instability and fall risk (Awad et al., 2017; Sloot et al., 2023). The absence of heel rocker function, often observed in a flat-foot or forefoot initial contact, is common in a dropfoot gait pattern and negatively affects weight transfer and acceptance during walking (Perry and Burnfield, 2010; Nolan and Yarossi, 2011). The assistance provided by the exoskeleton in the swing phase and just after initial contact shows promise to facilitate the restoration of the heel rocker function. Specifically, in this pilot group, it adequately positioned the foot at initial contact in the sagittal and frontal planes and controlled the initial plantarflexion. The normalized frontal plane ankle angle at initial contact can also potentially reduce the risk of ankle sprains during weight acceptance (DeMers et al., 2017).

A tendency to reduce muscle activation has been observed in previous studies of walking with exoskeletons (Reinkensmeyer et al., 2009; Awad et al., 2020). In the present study, muscle activation of the tibialis anterior in the swing phase was similar in the PowExo and the NoExo conditions, suggesting that the exoskeleton encourages some active muscle recruitment in the assisted muscle groups. This aligns with a previous study that reported sustained plantarflexor activation with an active ankle exoskeleton (Sloot et al., 2023). This observation, of course, only describes the exoskeleton’s immediate effects; further longitudinal studies are needed to validate whether this preservation of muscle activation persists with long-term use.

Improvements in holistic gait quality are also important for evaluating the effects of an intervention or assistive device (Yul Shin et al., 2020 Shin et al, 2022). In this study, we studied step length asymmetry, as it represents a holistic effect of the local assistance at the ankle, and observed that it decreased with the exoskeleton, which suggests the potential of the exoskeleton to improve broader aspects of gait quality.

A goal of this study was to evaluate whether the exoskeleton structure itself had any effect on gait, independent of the assistance it provides. To isolate this effect, the UnpowExo condition was performed with the cables slackened, thereby minimizing the influence of the inherent inertia and friction forces in the actuation system. Only slight differences in any gait metrics were found between the NoExo and the UnpowExo conditions, suggesting that the powered exoskeleton’s effects on gait are due to the assistance rather than to the passive properties.

Subjective feedback gathered from the two questionnaires focused on the exoskeleton’s effectiveness, comfort, and passive characteristics such as dimension, weight, and stability, and suggested that the exoskeleton largely meets key performance expectations and has the potential for user acceptance. These reported scores were also in line with other novel ankle exoskeletons for people post-stroke (de Miguel-Fernández et al., 2022). However, one participant provided relatively low scores in comfort-related items. This participant expressed discomfort due to the pressure of the calf wrap after prolonged wear, exacerbated by the presence of the EMG sensor underneath. Furthermore, while scores reflect overall satisfaction with the device’s effectiveness, participants expressed different levels of perceived assistance with the 10 assistive profiles, which was expected; further study to individualize assistance profiles is warranted, as is a study of whether improved objective gait metrics and subjective experience agree.

There are, of course, some limitations. This feasibility study included a small sample size of five participants, and the selection criteria were restrictive, making the findings not generalizable to a broader population. The evaluation of the exoskeleton’s effectiveness was conducted once in a controlled laboratory setting; this environment allowed for precise measurements and reduced confounding variables, but cannot fully reflect real-world conditions. Future studies should include a larger and more heterogeneous study population, several adaptation and experimental sessions, and various environments and terrains to verify and extend these preliminary results to daily life contexts. The assistive profiles used in this study were within a span introduced in a prior study. While they suggest a relatively promising effect, optimal assistance is likely individual; even in this relatively homogenous pilot group, the varied responses to the exoskeleton assistance suggest the importance of tailoring exoskeleton assistance to individual needs. Future work should focus on exploring personalized assistance strategies that fulfill clinically relevant objectives.

## 5. Conclusion

In this study, we demonstrated the feasibility of the newly developed soft ankle exoskeleton designed to correct ankle and foot position in two planes in persons with dropfoot gait after a stroke. With the assistive exoskeleton, immediate improvements in counteracting dropfoot with or without excessive inversion and in holistic gait patterns were observed in the five participants. These findings, coupled with subjective satisfaction, encourage further pursuit on a larger sample of participants, over a longer time, and on varied terrains.

## Figure 1. Long description

The figure has two parts. Panel A shows a person wearing a cable‑driven ankle exoskeleton. A backpack contains the actuation system, control unit, and battery. Two Bowden cables run from the backpack down the leg to the forefoot of the shoe. A textile calf wrap anchors the cable path, and sensors and a foot switch are attached near the shoe. Panel B presents a schematic of the three‑level controller. The high‑level controller detects gait phases and switches between stance and swing. The mid‑level controller includes a torque‑profile generator and a force‑free controller that determines desired cable tension. The low‑level controller tracks the commanded current to drive the actuator. Arrows indicate information flow between the three layers.

Navigate back to Figure 1..

## Figure 2. Long description

The figure shows a line graph of assistive dorsiflexion torque over a full gait cycle from 0% to 100%. The vertical axis represents torque in N·m/kg, and the horizontal axis represents gait cycle percentage. A pink line shows the medial motor torque profile, and a red line shows the lateral motor torque profile. A blue dashed line represents an example combined torque profile from both motors. A gray shaded region highlights the possible range for the peak torque tp. Small foot icons mark key gait events: foot contact at the start of the cycle, toe‑off near mid‑cycle, and foot contact again at the end. The curves illustrate how torque contributions from the medial and lateral motors vary throughout the gait cycle and how their sum produces the overall assistive torque.

Navigate back to Figure 2..

## Table 1. Long description

The table presents clinical and functional information for five participants (P01–P05). Columns include paretic side, time since stroke onset in months, walking aids used, Fugl‑Meyer Assessment lower‑extremity motor and sensory scores, Modified Ashworth Scale (MAS) spasticity scores for three ankle‑related muscles (soleus, gastrocnemius and ankle supinator), and passive ankle range of motion (maximum dorsiflexion and plantarflexion). P01 has left‑side paresis, 21 months post‑stroke, uses no walking aid, and has motor and sensory FMA‑LE scores of 26 and 7. MAS scores are (0, 0, 0), and ankle range of motion is 0° -30° . P02 has left‑side paresis, 60 months post‑stroke, uses an AFO, and has motor and sensory scores of 21 and 7. MAS scores are (3, 3, 1), with ankle range from –5° to 30°. P03 has left‑side paresis, 58 months post‑stroke, uses an AFO, and has motor and sensory scores of 25 and 9. MAS scores are (2, 0, 0), and ankle range is –2° to 45°. P04 has left‑side paresis, 24 months post‑stroke, uses an AFO, and has motor and sensory scores of 25 and 9. MAS scores are (0, 0, 0), with ankle range from 20° to 30°. P05 has right‑side paresis, 39 months post‑stroke, uses a Dictus band, and has motor and sensory scores of 29 and 12. MAS scores are (0, 2, 0), and ankle range is 5° to 60°.

Navigate back to Table 1..

## Figure 4. Long description

The figure contains five line graphs, one for each participant (P01–P05), showing ankle joint angles over a full gait cycle from 0% to 100%. The vertical axis represents plantarflexion (positive) and dorsiflexion (negative) angles in degrees. Each graph includes three conditions: NoExo (red), UnpowExo (green), and PowExo (blue). Across participants, differences between conditions appear more dorsiflexion angle in the swing phase and initial contact, with PowExo generally showing altered angle trajectories compared to NoExo and UnpowExo.

Navigate back to Figure 4..

## Figure 5. Long description

The figure contains four bar graphs labeled A–D, each comparing three walking conditions: NoExo (red), UnpowExo (green), and PowExo (blue). In all panels, bars represent median values across five participants, and individual markers (circles, squares, triangles, diamonds, inverted triangles) show each participant’s data. Panel A shows foot clearance height in millimeters, Panel B shows peak dorsiflexion angle during swing in degrees, Panel C shows maximum inversion angle during swing in degrees, and Panel D shows tibialis anterior EMG amplitude during swing, normalized to each participant’s peak activation.

Navigate back to Figure 5..

## Figure 6. Long description

The figure contains three bar graphs labeled A–C, each showing foot and ankle position metrics at initial contact for five participants under three conditions: NoExo (red), UnpowExo (green), and PowExo (blue). Bars represent median values across participants, and individual markers connected by thin lines show each participant’s measurement. Panel A displays foot inclination angle in degrees, Panel B shows ankle dorsiflexion angle in degrees, and Panel C presents foot inversion angle in degrees.

Navigate back to Figure 6..

## Figure 7. Long description

The bar chart has three vertical bars from left to right labeled NoExo, UnpowExo, and PowExo on the x axis. The y axis is labeled Step length asymmetry in percent, ranging from 0 to 30. The NoExo bar is dark red and reaches about 16 percent, UnpowExo is light green and slightly higher at about 18 percent, and PowExo is dark blue and lower at about 11 percent. Overlaid on each bar are five individual markers representing participants P01 to P05, each with a unique shape: circle, right triangle, square, upward triangle, and diamond. Lines connect each participant’s data point across the three conditions, showing a general trend of decreasing asymmetry from NoExo and UnpowExo to PowExo for most participants. The legend at the top right identifies marker shapes for each participant.

Navigate back to Figure 7..

## Figure 8. Long description

The figure contains two panels summarizing questionnaire responses about participants’ satisfaction with the exoskeleton. Panel A is a radar chart with five axes: stability and security, weight, comfort, effectiveness, and dimensions. Each axis ranges from 0 to 5. Five colored lines represent individual participants (P01–P05). The chart shows how each participant rated the exoskeleton across the five dimensions. Panel B contains six box plots, each corresponding to a question scored from 0 to 10. The questions address: ease of walking with the exoskeleton, discomfort, skin problems, pain, perceived benefit of the exoskeleton, and perceived benefit of the participant’s everyday ankle orthosis. Each box plot shows the distribution of responses across participants, with the white circle marking the median and the box edges representing the 25th and 75th percentiles.

Navigate back to Figure 8..
